# Clinical performance comparison between lithium disilicate and hybrid resin nano-ceramic CAD/CAM onlay restorations: a two-year randomized clinical split-mouth study

**DOI:** 10.1007/s10266-023-00841-w

**Published:** 2023-08-05

**Authors:** Amr Hassan, Kareem Hamdi, Ashraf I. Ali, Walid Al-Zordk, Salah Hasab Mahmoud

**Affiliations:** 1 Operative Dentistry Department, Faculty of Dentistry, Mansoura University, Mansoura, Egypt; 2 Operative Dentistry Department, Faculty of Dentistry, Zagazig University, Zagazig, Egypt; 3 Fixed Prosthodontics Department, Faculty of Dentistry, Mansoura University, Mansoura, Egypt; 4 Kareem Dental Clinic, El Guesh street, Mansoura, 35516 Egypt; 5 Operative Department, Faculty of Dentistry, Horus University, New Damietta, Egypt

**Keywords:** IPS e.max, Lithium disilicate, Hybrid ceramic, Resin nano-ceramic, Onlays, Indirect ceramic restorations, Partial coverage

## Abstract

A total of 20 lithium disilicate glass–ceramics (IPS e.max CAD, Ivoclar Vivadent) and 20 resin nano-ceramic (Voco Grandio Blocks) onlay restorations were performed in 20 patients using a split-mouth design to compare the two-year clinical performance of lithium disilicate and resin nano-ceramic onlay restorations. Both restorations were evaluated at baseline, one-year, and two-year clinical follow-ups based on the modified United States Public Health Service (USPHS) criteria. Chi-square and Fisher’s exact tests showed no statistically significant difference between Voco Grandio and IPS e.max ceramic restorations for all evaluated parameters during the different follow-up periods (*p* > 0.05). Cochrane’s and MC-Nemar’s tests indicated statistically significant differences regarding color match within the Voco Grandio group. They also indicated statistically significant differences in marginal discoloration, marginal adaptation, surface texture, and postoperative hypersensitivity within both ceramic material groups (*p* < 0.05). Kaplan–Meier curve indicated that the survival rate of both ceramic materials was 90%. After two years of clinical service, IPS e.max CAD and Voco Grandio onlay restorations exhibited similar clinical performance.

## Introduction

Advances in restorative dentistry have gained significant importance for meeting patient expectations for posterior restorations. Indirect ceramic restorations offer a conservative alternative to treating large cavities and can address many of the limitations associated with direct composite restorations [1–3]. Computer-aided design/computer-aided manufacturing (CAD/CAM) technology has gained popularity, enabling the fabrication of restorations that are directly chairside within a single appointment. This approach has been made possible by advancements in CAD/CAM systems and intraoral scanners, which have revolutionized restorative dentistry by overcoming the constraints of conventional impression techniques and manufacturing methods. Furthermore, introducing various indirect ceramic blocks with innovative microstructures and compositions has resulted in significant divergence among materials regarding their mechanical and biological properties, bond stability, and long-term clinical performance [4].

In terms of composition and microstructure, ceramic blocks can be categorized as oxide ceramics (such as zirconium oxide and aluminum oxide), glass–ceramics (including lithium disilicate and leucite-reinforced glass–ceramics), hybrid ceramics, and resin matrix composite blocks [5, 6]. Recent advancements in materials have sparked extensive research within the dental community to comprehensively evaluate the properties and clinical performance of these materials. Consequently, numerous laboratory studies [7–10], clinical studies [11–13], and systematic reviews have been published on this subject [5, 14–17]. Despite the favorable mechanical properties of glass–ceramic materials, they are prone to fracture and wear of the opposing teeth, as indicated by available evidence [5]. Therefore, there has been growing interest in developing materials that combine the desirable characteristics of glass–ceramics and composite resins.

Lithium disilicate ceramic was first introduced in 1998 with the launch of IPS Empress 2 (Ivoclar Vivadent, Schaan, Principality of Liechtenstein), specifically designed for use with press technology. In 2005, IPS Empress 2 was replaced with the updated versions known as IPS Empress CAD and IPS e.max CAD. The current IPS e.max CAD variant can be identified by its color-coded blue block, which consists of a metasilicate state comprising 40% platelet-shaped lithium metasilicate crystals embedded in a glass matrix [18, 19]. Numerous studies have demonstrated the satisfactory clinical performance of lithium disilicate ceramic materials in restorative dentistry [11, 13, 18, 20, 21].

Resin nano-ceramic (RNC) materials with promising mechanical properties have recently emerged, combining the advantages of ceramics and composite resins. The elastic nature of the RNC allows easy intraoral milling and repair [4]. Compared with the complete replacement required for failed conventional ceramic materials, the intraoral repair is considered a more conservative and cost-effective approach with a lower risk of pulpal damage [4]. Wear resistance is another crucial factor that influences clinical performance. Wear refers to the natural degradation of restorative materials caused by abrasive procedures in the oral environment [22]. Various published studies indicate that RNC materials exhibit lower wear resistance than conventional glass–ceramic materials [23, 24]. However, another study suggested that RNCs may demonstrate wear resistance comparable to glass–ceramic materials [25].

Furthermore, increasing evidence supports the acceptable clinical performance of RNCs compared with conventional glass–ceramics [5, 12]. The contradiction in the aforementioned evidence could be attributed to variations in the organic matrix and inorganic filler particles among different brands [26]. Voco Grandio blocks (VOCO GmbH, Cuxhaven, Germany) are an example of an RNC material comprising 86% inorganic filler embedded in a polymer matrix. These blocks exhibit hardness and modulus of elasticity (18.28 GPa), similar to those of natural tooth structures [17, 27]. After an extensive search for published evidence, numerous studies evaluating the clinical performance of glass–ceramic restorations have been conducted [11, 13, 28–35]. However, limited information exists regarding the clinical performance of RNC restorations [12, 13, 36]. Therefore, the objective of the present study was to fill this gap by comparing the clinical performance of machinable RNC and lithium disilicate ceramic materials using the modified United States Public Health Service (USPHS) criteria over a 2-year follow-up period. The research hypothesis proposed that there would be no significant difference in the performance of restorations fabricated with IPS e.max CAD or RNC (Voco Grandio) after a two-year clinical follow-up.

## Materials and methods

### Ethical approval

This clinical study was approved by the Dental Research Ethics Committee at the Faculty of Dentistry, Mansoura University (approval number A20090419) and registered in the clinical trial registration database (www.clinicaltrials.gov) under identification number NCT05556551. The research procedure was thoroughly explained to the participants, who were provided with written information about the study and signed a written informed consent form. This study was a randomized controlled clinical trial with a split-mouth design. To ensure blinding, neither the participants nor the evaluators knew the dental materials used. This resulted in a double-blind study that adhered to the CONSORT 2010 statement for reporting trials [37]. The study commenced on May 20, 2019 and was officially completed on July 20, 2021.

### Sample size calculation

The sample size was calculated using the G*Power statistical software (version 3.0.10, Franz Faul, Universitat Kiel, Germany). During the sample size calculation, a statistical power of 80%, an α error of 5%, and a predicted sample loss of 20% at the end of the study were considered. Based on a previously published study [13], it was determined that 20 patients would be the appropriate sample size for this study. Within each group, IPS e.max CAD (Ivoclar Vivadent) and Voco Grandio (VOCO, Cuxhaven, Germany) twenty patients received one restoration of the respective group in the molar/premolar region on one side, and one on the other side.

### Patient recruitment and eligibility criteria

Participants were recruited through social network advertisements and posters displayed in an outpatient clinic at Faculty of Dentistry, Mansoura University. After 2 months of advertising, 34 patients were recruited for the study. Following inclusion and exclusion criteria, 20 patients aged between 25 and 40 years were enrolled in this study. Eligible participants had two posterior teeth with significant defects in dental hard tissue, characterized by an occlusal cavity width greater than one-third of the bucco-lingual width in the molar/premolar region on both sides. Table 1 presents the inclusion and exclusion criteria in detail.

**Table1:** Inclusion and exclusion criteria for study patients and teeth

Inclusion criteria	Exclusion criteria
The patients had to show two posterior teeth with large defects on dental hard tissue (occlusal cavity width > 1/3 of the width in bucco-lingual direction) in premolar/ molar region in both sides Good/acceptable oral hygiene level (patients with low, and moderate caries risk using CAMBRA risk assessment method were included in the study) Good/acceptable periodontal health (periodontal screening index < 2 and papilla bleeding index < 35%) Positive sensitivity test to cold of all teeth intended to be treated Opposing and neighboring tooth are present and sound Possibility of rubber dam isolation	Peridontits (periodontal screening index > 2), and Papilla bleeding index > 35% Abnormal occlusal forces (bruxism, parafunctional forces) Alcohol and drug abusers, pregnant females, and general systemic diseases that may lead to reduction of life expectations

*CAMBRA* caries management by risk assessment

### Restorative procedure

The clinical restorative procedure was conducted by a third partner (WI), a postgraduate student who was not directly involved in the study. The restorative procedures were conducted under the supervision of an experienced restorative dentist (YS) who did not participate in the study. Throughout the treatment process, an experienced restorative dentist (YS) closely examined all steps, including diagnosis, cavity preparation, caries excavation, buildup, undercut blockage, impression, optical scanning, digital workflow, cementation, and final finishing of the adhesively luted restorations. Cavity preparation followed established principles for indirect adhesive restorations [38]. Local anesthesia was administered during cavity preparation, and all cavity walls diverged by approximately 10–15°. The occlusal cavity width exceeded one-third of the bucco-lingual width, internal lines and angles were rounded, and the pulpal floor depth was maintained at 1.5–2 mm to ensure the appropriate thickness of the restoration. A caliper was used to measure the base of each cusp to determine which cusps needed to be covered during tooth preparation [38]. Functional cusps were reduced by 2 mm, whereas non-functional cusps were reduced by 1.5 mm. Cavity preparations were conducted using a tapered, rounded stone bur (847KR, G&Z, Austria) with no bevel enamel margin, utilizing a high-speed handpiece with coolant. Subsequently, the cavities were refined using a fine stone bur (XF878, G&Z, Austria), rubber cups, and tips with a low-speed handpiece. All preparations were performed under magnification with magnifying loupes (X 3.5, Amtech, USA).

After preparation, impressions were obtained using an Elite HD with low viscosity (Zhermack, Italy). Following the impressions, provisional restorations were created using light cure and eugenol-free temporary fillings (NextTempLC, MetaBiomed, Korea) and placed in the prepared cavities. The impressions were poured and sent to the dental lab office, where the same dental technician manufactured all restorations. The models were scanned, and a milling machine (imes-icore CORiTEC 350i, imes-icore GmbH, Eiterfeld, Germany) was used to mill the CAD/CAM blocks. All restorations were designed by an experienced dental technician using ExoCAD software under the supervision of an experienced restorative dentist (YS). The occlusal morphology suggested by the software was adjusted to align with the existing occlusion during maximum intercuspation and lateral excursion. The internal cement gap for all the restorations was set at 100 μm.

### Randomization and allocation concealment

Random allocation was achieved by drawing lots, with the procedure carried out by a third partner (WI) in the presence of a supervising, experienced dentist (YS). One tooth was assigned for the insertion of a lithium disilicate (IPS e.max CAD) onlay restoration, whereas the other tooth was designated for the insertion of a hybrid RNC (Voco Grandio blocks) onlay restoration [39]. Random allocation was performed after patient recruitment and before cavity preparation. Table 2 provides detailed technical information on both materials.

**Table 2:** Materials used in the study

Materials	Specification	Composition	Manufacture	Lot no.
Lithium Disilicate Glass Ceramic blocks (IPS e.max CAD)	Glass ceramic CAD/ CAM blocs	Sio_2_, Lio_2_, K_2_o, P_2_o_5_, Zro_2_, Zno Other oxides, coloring oxides	Ivoclar Vivadent	Z014XY
Hybrid Resin Nano-Ceramic blocks (Voco Grandio)	Hybrid ceramic CAD/CAM blocs	86% inorganic fillers, nano ceramic particles	VOCO Cuxhaven, Germany	2050495
Duo Link Universal resin cement	A dual cured adhesive resin cement	Base: Bisphenol-A glycidyl dimrthacrylate, uncured dimethacrylate monomer, glass filler Catalyst: phosphoric acidic monomer, glass fillers	Bisco: Schaumburg, IL, USA	2100003943
Silane	Porcelain primer	Ethanol, Silane coupling agent	Bisco Inc., Schaumburg, IL, USA	1200004083

After milling, the ceramic restorations were tested for fit in situ using try-in silicone (Fit Checker, GC Corporation, Tokyo, Japan). Marginal adaptation was assessed using a dental probe with a tip diameter of approximately 100 μm (EXS9, Hu-Friedy, Chicago, USA), while proximal contact was evaluated using dental floss. If necessary, adjustments were made using a fine diamond finishing bur (XF471, G&Z, Austria), and the restorations were sent back to the dental lab for an additional glazing cycle.

### Adhesive luting of restorations

Before the adhesive luting of the restorations, a rubber dam was applied to isolate the treatment area, and the respective teeth were cleaned using a mixture of pumice and water. They were then rinsed with a water spray and lightly air-dried. The timeline for patients’ enrollment in the study following inclusion and exclusion criteria, randomization, cavity preparation, temporization, try-in, and adhesive luting of the final restoration was 2 weeks; the first patient entered the study, and the last patient completed the procedures within this timeframe.

The manufacturer’s instructions were followed for the surface pretreatment of the restorations. The inner surface of the IPS e.max CAD restorations was etched with 9.5% hydrofluoric acid solution (Porcelain Etchant Bisco, Schaumburg, IL, USA) for 20 s. The samples were then rinsed with water for 20 s and air-dried for 5 s. A thin silane coupling agent layer (Bisco Inc., Schaumburg, IL, USA) was applied and left for 1 min, followed by air drying.

In the case of RNC restorations (VOCO Grandio), the inner surfaces were subjected to sandblasting using 30-micron alumina particles for 20 s at a pressure of 2 bar. Subsequently, the surfaces were cleaned with water and dried. A silane coupling agent was applied and allowed to air dry.

The enamel margins were selectively etched using 37% phosphoric acid (3M ESPE, St. Paul, MN, USA) for 30 s. The etched surfaces were then rinsed with a water spray and gently air-dried until they were chalky white. All Bond Universal adhesive (Bisco, Schaumburg, IL, USA) was applied to the dentin and enamel surfaces and rubbed for 20 s. The adhesive layer was then slowly air-thinned for 5 s to allow for solvent evaporation. Finally, the adhesive was light-cured for 20 s.

For cementation, dual-cure resin cement (Duo Link Universal, Bisco, Schaumburg, IL, USA) in a universal shade was used, following the manufacturer’s instructions. To facilitate the handling and insertion of the restoration into the prepared cavity, a microbrush was bonded to the occlusal surface of the restoration using Liquidam (Bleaching Dam, White Smile, Germany).

Excess cement in the contact areas was removed using a microbrush and dental floss. Each restoration surface (occlusal, buccal, and palatal) was light-cured for 60 s. After removing the rubber dam, the occlusal surface was adjusted using fine diamond burs (XF878, G&Z, Austria) and checked for high points using articulator paper.

Finally, the glass–ceramic and RNC restorations were polished using polishing points and paste (Diapol Kit RA305, Eve, Naples, Florida, USA).

Patients were contacted via telephone in the first week after the initial appointment to assess for postoperative hypersensitivity. A criterion-referenced rating scale was used to evaluate the level of postoperative hypersensitivity experienced by patients [36]. This telephone interview served as a follow-up procedure to minimize the risk of recall loss, as the patients did not need to return to the clinic. During the telephone interview, patients were instructed to schedule a clinic visit if they experienced intolerable postoperative hypersensitivity or noticed premature contact with their restorations. Patients were also instructed to contact us via telephone if they experienced any problems like fractures or debonding.

The sensitivity criteria used in the assessment were as follows:

- Score 1: No sensitivity was experienced at any time.
- Score 2: Slight sensitivity is experienced occasionally but is not uncomfortable.
- Score 3: Moderate sensitivity was experienced intermittently and was noticeably uncomfortable.
- Score 4: Severe discomfort was routinely noted with cold or pressure stimulation.

Clinical evaluations were made at baseline (after adhesive luting of restoration), one-year, and two-year follow-up appointments by two independent evaluators (AG and RW) who were not involved in the study. They had undergone preliminary calibration procedure using written criteria based on modified United States Public Health Service (USPHS) criteria for margin discoloration, aesthetic anatomical form, color match, marginal adaptation, secondary caries, debonding and fracture (Table 3). In the case of disagreements during the clinical evaluation process, the evaluators discussed their differences and reached a consensus judgment before dismissing the patient. This approach ensured that any disagreement was resolved through consensus between evaluators.

**Table 3:** The modified USPHS criteria

Category	Rating
Color match	
Tooth and restoration have an ideal color match; restoration can be difficult to distinguish	ALFA
Readily perceptible mismatch in color; general match	Bravo
Obvious mismatch in color between tooth and restoration; unacceptable	Charlie
Marginal discoloration	
No evidence of margin discoloration	ALFA
Surface stain along less than 50% of the exposed margin	Bravo 1
Surface stain along greater than 50% of the exposed margin	Bravo 2
Penetrating discoloration of exposed margin	Charlie
Aesthetic anatomic form	
Restoration is continuous with the existing anatomic form	ALFA
Restoration is discontinuous with existing anatomic form, and missing material is not sufficient in size to expose dentin	Bravo
Restoration is discontinuous with existing anatomic form and missing material sufficient in size to expose dentin	Charlie
Recurrent caries	
No evidence of caries	ALFA
Evidence of recurrent caries at the crown margin. The carious lesion is repairable/not compromise the crown	Bravo
Evidence of recurrent caries at the crown margin. The carious lesion is not repairable/crown and requires replacement	Charlie
Margin adaptation	
No evidence of crevice formation along the cavosurface margin; the explorer does not catch when its tip moves across the margin	ALFA 1
Margin is detectable along less than 50% of cavosurface margin and less than 1 mm in depth	ALFA 2
Margin is detectable along more than 50% of the cavosurface margin and less than 1 mm in depth	ALFA 3
Evidence of penetrable crevices along less than 50% of cavosurface margin and greater than 1 mm in depth	Bravo 1
Evidence of penetrable crevices along greater than 50% of the cavosurface margin and greater than 1 mm in depth	Bravo 2
Evidence of crevice formation exposing dentin to the axial or pulpal floor	Charlie
Restoration fracture	
No evidence of onlay fracture ALFA	ALFA
Evidence of onlay fracture confined to less than 50% of the occlusal isthmus width; the fractured piece is not mobile	Bravo
Evidence of onlay fracture extending more than 50% of the occlusal isthmus width; fractured pieces are not mobile	Charlie
Fracture of onlay with mobile pieces	Delta
Postoperative hypersensitivity	
No sensitivity is experienced at any time	ALFA
Slight sensitivity is experienced occasionally but is not uncomfortable	Bravo
Moderate sensitivity is experienced intermittently and is noticeably uncomfortable	Charlie
Severe discomfort is noted routinely with cold or pressure stimulation	Delta

### Clinical examination

The restorations were assessed using the modified USPHS criteria for the clinical evaluation of dental restorations. Mirrors, probes, and dental loupes (X 3.5) (Amtech, USA) were used to thoroughly examine the restorations. In addition to clinical examination, digital periapical radiographs were obtained using an I-sensor digital radiography system (Guilin Woodpecker, China).

To document the clinical appearance of the restorations, digital clinical photographs were taken using a digital single-lens reflex (DSLR) camera (Canon EOS 1200D, Tokyo, Japan) with a 100-mm F macro lens (Tukina, Japan). The camera settings were ISO 100, aperture F 25, and a ring flash in the manual mode set to ¼ power (Canon, Tokyo, Japan). These photographs were taken at baseline, 1-year, and 2-year follow-up appointments to track any changes in the appearance of the restorations over time (Fig. 3).

### Statistical analysis

Statistical analysis was carried out using the Chi-Square test for comparison of two restorative groups, the Fischer exact test was used as a correction for the Chi-square. Cochrane’s test was used to determine if there are differences within the same group, while the MC-Nemar’s test was used for comparison between baselines and different follow-up periods (Table 4). The Kaplan–Meier test was used to calculate the functional survival rate (Fig. 2).

**Table 4:** summarizes the results and ratings obtained for both ceramic materials at baseline, one- and 2-year recalls, according to modified USPHS criteria [number of restorations (*N*) and percentages (%)]

	Time of assessment	Voco Grandio group *N* (%)	IPS-emax ceramic group *N* (%)	*P* value*
Post-operative Sensitivity	Baseline ALFA BRAVO	*N* = 20 14 (70.0)^ab^ 6 (30.0)	*N* = 20 14 (70.0)^ab^ 6 (30.0)	1.0
	12 months follow up ALFA BRAVO	*N* = 18 18 (100)^a^ 0	*N* = 18 16 (88.9)^a^ 2(11.1)	0.486
	24 months follow up ALFA BRAVO	*N* = 18 18 (100)^b^ 0	*N* = 18 17 (94.4)^b^ 1(5.6)	1.0
*P* value^#^		0.007*	0.015*	
Anatomic form	Baseline ALFA BRAVO	*N* = 20 20 (100) 0	N = 20 20 (100) 0	….
	12 months follow up ALFA BRAVO	*N* = 18 18 (100) 0	*N* = 18 17 (94.4) 1 (5.6)	1.0
	24 months follow up ALFA BRAVO	*N* = 18 18 (100) 0	*N* = 18 17 (94.4) 1 (5.6)	1.0
*P* value^#^		1.0	0.368	
Color match and translucency	Baseline ALFA BRAVO	*N* = 20 17 (85) 3 (15)	*N* = 20 16 (80) 4 (20)	1.0
	12 months follow up ALFA BRAVO	*N* = 18 15 (83.3)^a^ 3(16.7)	*N* = 18 13 (72.2) 5 (27.8)	0.691
	24 months follow up ALFA BRAVO	*N* = 18 11 (61.1)^a^ 7 (38.9)	*N* = 18 11 (61.1) 7 (38.9)	1.0
*P* value^#^		0.018*	0.174	
Marginal discoloration	Baseline ALFA BRAVO	*N* = 20 20 (100)^a^ 0	*N* = 20 20 (100)^ab^ 0	….
	12 months follow up ALFA B	*N* = 18 15 (83.3)^b^ 3 (16.7)	*N* = 18 14 (77.8)^a^ 4 (22.2)	1.0
	24 months follow up ALFA BRAVO	N = 18 11(61.1)^ab^ 7(38.9)	N = 18 11(61.1)^b^ 7(38.9)	1.0
*P* value^#^		0.005*	0.01*	
Marginal adaptation	Baseline ALFA BRAVO	*N* = 20 20 (100)^a^ 0	*N* = 20 20 (100)^ab^ 0	….
	12 months follow up ALFA BRAVO	N = 18 15 (83.3) 3 (16.7)	N = 18 13 (72.2) ^a^ 5 (27.8)	0.423
	24 months follow up ALFA BRAVO	*N* = 18 12 (66.7)^a^ 6 (33.3)	*N* = 18 11 (61.1)^b^ 7 (38.9)	0.729
*P* value^#^		0.01*	0.008*	
Surface texture	Baseline ALFA BRAVO	*N* = 20 20 (100) 0	*N* = 20 20 (100)^a^ 0	….
	12 months follow up ALFA BRAVO	*N* = 18 15 (83.3)^a^ 3 (16.7)	*N* = 18 15 (83.3) 3 (16.7)	1.0
	24 months follow up ALFA BRAVO	*N* = 18 13 (72.2)^a^ 5 (27.8)	*N* = 18 12 (66.7)^a^ 6 (33.3)	0.717
*P* value^#^		0.02*	0.01*	
Recurrent caries	Baseline ALFA BRAVO	*N* = 20 20 (100) 0	*N* = 20 20 (100) 0	….
	12 months follow up ALFA BRAVO	*N* = 18 18 (100) 0	*N* = 18 16 (88.9) 2 (11.1)	0.486
	24 months follow up ALFA BRAVO	*N* = 18 17 (94.4) 1 (5.6)	*N* = 18 16 (88.9) 2 (11.1)	1.0
*P* value^#^		0.368	0.264	
Fracture	Baseline ALFA BRAVO	*N* = 20 20 (100) 0	*N* = 20 20 (100) 0	….
	12 months follow up ALFA BRAVO	*N* = 18 18 (100) 0	*N* = 18 18 (100) 0	….
	24 months follow up ALFA BRAVO	*N* = 18 18 (100) 0	*N* = 18 18 (100) 0	….
*P* value^#^		…	…	
Debonding	Baseline ALFA BRAVO		*N* = 20 20 (100) 0	
	12 months follow up ALFA BRAVO		*N* = 18 18 (100) 0	
	24 months follow up ALFA BRAVO		*N* = 18 18 (100) 0	
*P* value^#^			……	

*Chi-square and Fischer exact test

^#^Cochrane test, similar superscripted letters denote significant difference between groups within same column by MC-Nemar test

## Results

**Fig. 1:**
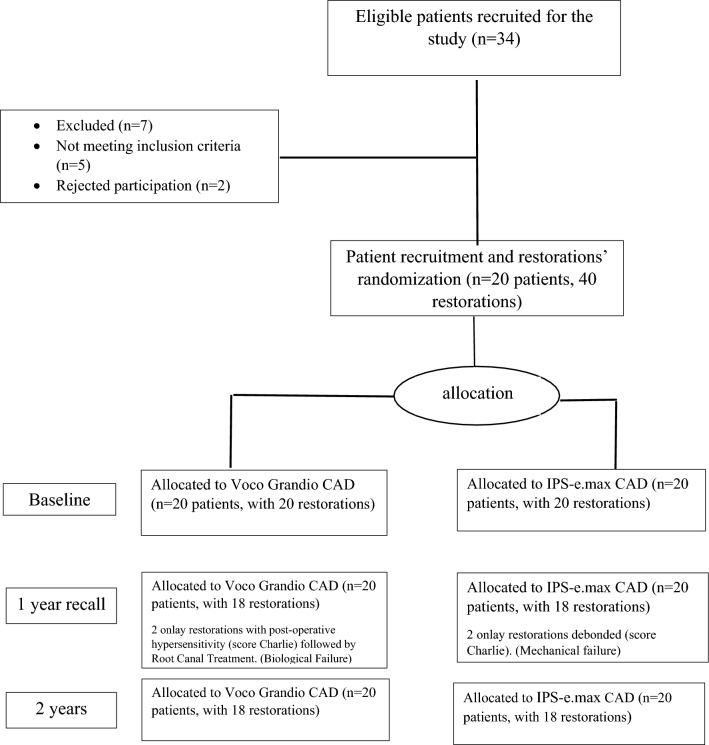
CONSORT flow chart

This study included 20 patients and 40 onlay restorations placed in the posterior molar/premolar regions. The recall rates of all patients in the first week, after one year, and after two years were 100%. In the 1-year clinical follow-up, two IPS-e.max onlay restorations were debonded. Although these restorations were successfully rebonded, they were considered mechanically failed after 1-year clinical follow up. Furthermore, all restorations remained clinically acceptable after the 2-year clinical follow-up period. Figure 1 presents a flow chart illustrating the participation of 20 patients in this study.

### Clinical evaluation

The percentage distributions of the scores based on the modified USPHS criteria, including postoperative hypersensitivity, aesthetic anatomic form, color match, surface texture, marginal adaptation, marginal discoloration, secondary caries, fracture, and debonding, are presented in Table 4 for the baseline and 1-year and 2-year clinical follow-up periods.

Fisher’s exact test was used to determine whether there were significant differences between the different ceramic materials in terms of all criteria during the different clinical follow-up periods (*p* > 0.05). Statistical analysis revealed no significant differences between the groups. Furthermore, the impact of the co-variables (premolar vs. molar) on the clinical performance of both ceramic restorations was assessed, and no significant differences were observed (*p* > 0.05).

Cochrane’s test was used to determine if there are differences within the same group, followed by the post hoc MC-Nemar’s test. The results indicated significant differences in color match within the Voco grandio group after 2- year clinical follow-up. The results also indicated significant differences in marginal discoloration, marginal adaptation, surface texture, and postoperative hypersensitivity within both ceramic material groups (Table 4).

No significant differences were observed between the different ceramic materials in terms of postoperative hypersensitivity at the different follow-up periods (*p* > 0.05). Additionally, significant differences were detected within both ceramic material groups in terms of postoperative hypersensitivity after a 2-year follow-up (*p* < 0.05).

Postoperative hypersensitivity resolved in all patients during the first week after adhesive luting of the restorations. However, two patients who received Voco Grandio restorations reported extended postoperative hypersensitivity lasting one month after adhesive luting. The same patients reported intolerable hypersensitivity after 1-year clinical follow-up, which was scored using the Charlie (*C*) score. Subsequently, root canal treatments were performed in these patients for these two teeth (one molar and one premolar). The two-root canal treated teeth were considered biologically failed after 1-year clinical follow- up.

Regarding the esthetic anatomic form, no significant differences were found between different ceramic materials at different clinical follow-up periods (*p* > 0.05). Additionally, there was no significant difference in the esthetic anatomic form within both ceramic material groups between the baseline and 2-year clinical follow-up. This suggests that the wear resistance of RNC restorations is comparable to that of lithium disilicate-based restorations (*p* > 0.05).

Regarding color matching, no significant differences were observed between the different ceramic materials at different clinical follow-up periods (*p* > 0.05). However, significant deterioration in color matching was noted within the Voco Grandio group after a 2-year clinical follow-up (*p* < 0.05).

In terms of marginal discoloration, no significant differences were observed between the different ceramic materials at different clinical follow-up periods (*p* > 0.05). Significant marginal discoloration was detected after 1-year clinical follow-up for IPS e.max group, while significant marginal discoloration was detected after 2-year clinical follow-up for Voco Grandio group (*p* < 0.05). Despite these findings, both types of restorations were considered clinically acceptable, and no Charlie (C) score was detected for either type of ceramic restoration.

Regarding marginal adaptation and surface texture, no significant differences were observed between the different ceramic materials at different clinical follow-up periods (*p* > 0.05). However, significant deteriorations were noted within both ceramic material groups regarding marginal adaptation and surface texture after 2-year clinical follow-up (*p* < 0.05).

In terms of fracture and secondary caries, no significant differences were found between the different ceramic materials at different clinical follow-up periods (*p* > 0.05). Additionally, no fractures or secondary caries were recorded in either type of ceramic materials after two years of clinical service, indicating a 100% success rate in this regard.

Regarding restoration retention, no significant differences were observed between the different ceramic materials at different clinical follow-up periods (*p* > 0.05). Furthermore, there were no significant differences in retention were detected within both ceramic material groups between the baseline and 1-year and 2-year clinical follow-ups (*p* > 0.05). Regarding IPS e.max ceramic restorations, two debonded restorations were recorded after one year of clinical follow-up. Although these two patients were asymptomatic prior to restorations’ debonding, no recurrent caries lesions were detected after debonding of restorations, the teeth were vital, and the restorations were successfully re-luted, these two restorations were considered mechanically failed after 1-year clinical follow-up.

### Kaplan–Meier survival rate

**Fig. 2:**
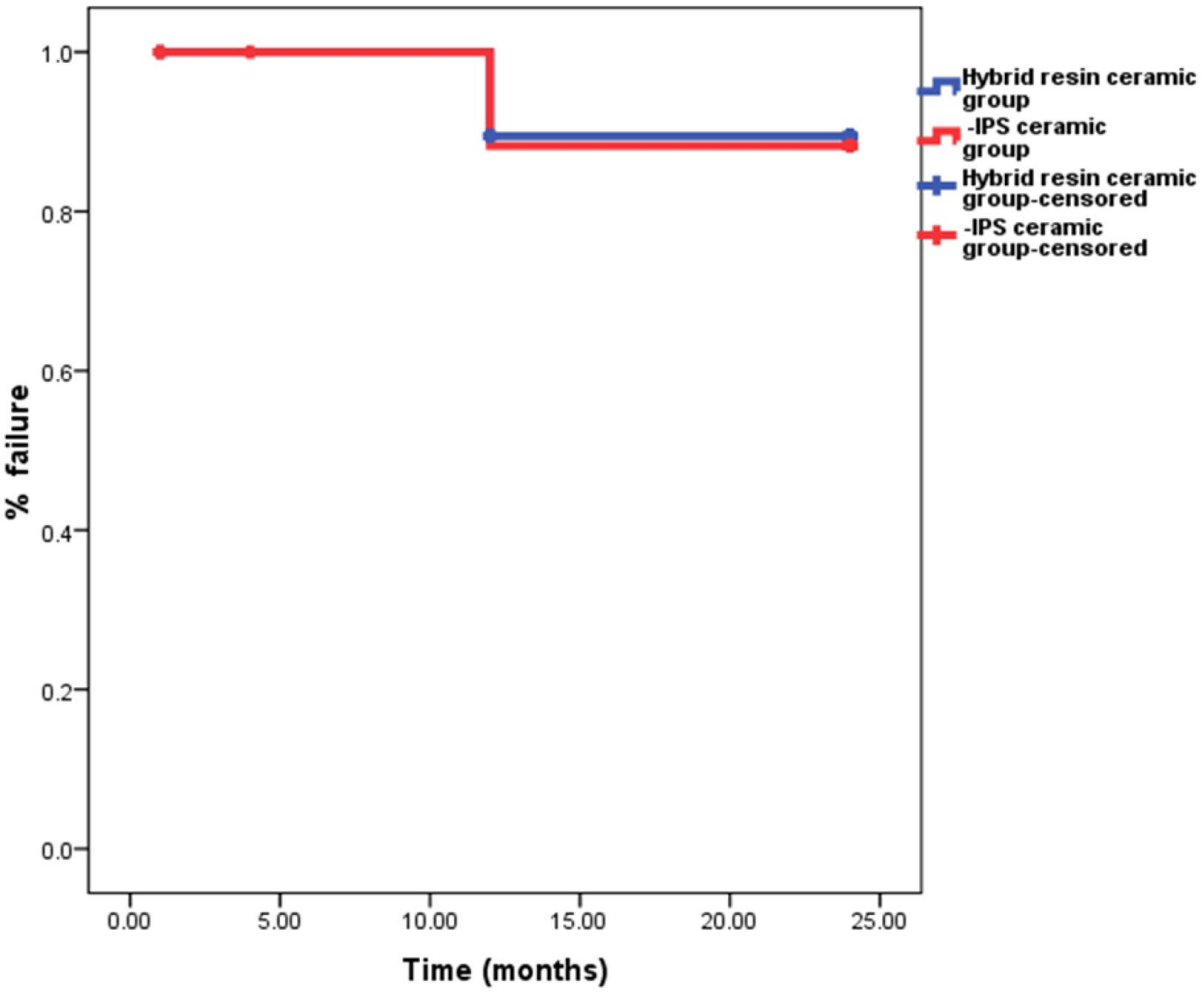
Kaplan-Miere curve showed percentage survival function of Voco Grandio (blue line) and IPS-e.max (red line) restorations during clinical follow-up periods. Survival function percentage was 90% for both ceramic materials after 2-year clinical follow-up

**Fig. 3:**
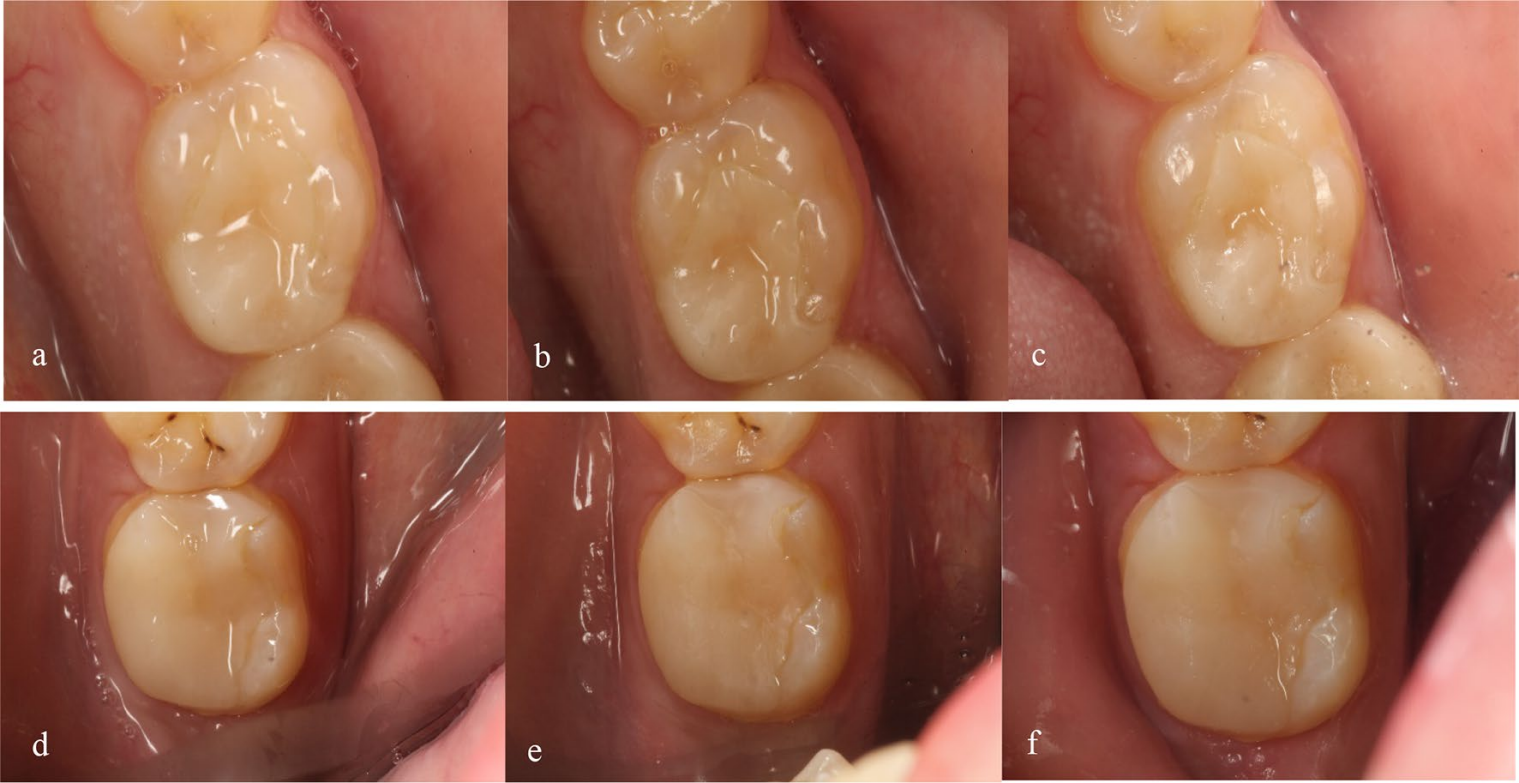
**a** IPS e.max CAD onlay restoration (baseline) **b** IPS e.max CAD after 1 year of follow-up, **c** IPS e.max CAD after 2-years of follow-up, **d** hybrid resin nano-ceramic (Voco Grandio) onlay restoration (baseline), **e** hybrid resin ceramic (Voco Grandio) onlay restoration after 1 year of follow-up, **f** hybrid resin nano-ceramic (Voco Grandio) onlay restoration after 2 years of follow-up

The survival rate of restorations after a 2-year clinical follow-up was 90% for both ceramic materials as depicted in Fig. 2. Statistical analysis revealed no significant differences between the two types of restorations during the different follow-up periods (*p* > 0.05) (Fig. 3).

## Discussion

The null hypothesis of the current study cannot be rejected, as no significant difference was observed in the clinical performance of restorations made from lithium disilicate (IPS e.max CAD) or RNC (Voco Grandio) after a 2-year clinical follow-up. A thorough review of the published literature revealed several clinical studies that assessed the clinical performance of glass–ceramics [11, 14, 28–35, 40–42]. However, there is a scarcity of data evaluating the clinical performance of RNC restorations, and few studies have directly compared different materials within the same study [5, 12, 13, 36]. Therefore, this study was designed to address this gap in literature.

Several factors contribute to the longevity of indirect restorations, including the risk of caries, occlusal load, and the clinician’s experience. Therefore, patients with a high caries risk index and improper occlusal patterns were excluded from the study [28, 43–45]. Therefore, this study used a split-mouth design to compare different restorations under the same clinical conditions. Restorative procedures were performed by a highly experienced operator (WI), a postgraduate student, under the direct supervision of an experienced restorative dentist (YS).

For both types of restoration, the cement gap space was set to 100 µm using Exocad software. In their laboratory study, Sokolowski, G., et al. [46] stated that utilizing a cement layer thicker than 25 µm, but not exceeding 200 µm, appears clinically acceptable. They further emphasized that a thicker cement layer (approximately 200 µm) generated significant contraction stresses, whereas a thinner cement layer (approximately 25 µm) resulted in high hygroscopic expansion stresses. These findings provide a clear rationale for selecting a cement gap of 100 µm for the present study.

Dual-cure resin cement is commonly favored for the cementation of indirect restorations because it can compensate for limited light transmission within the restoration. This enables complete polymerization even in areas that are difficult for light to penetrate, such as the bottom of the cavity [47, 48].

When comparing IPS e.max CAD and Voco Grandio restorations at baseline and after a two-year clinical follow-up, no significant differences were observed based on the UPSHS criteria. However, both materials exhibited notable changes in marginal discoloration, marginal adaptation, surface texture and postoperative hypersensitivity.

The marginal adaptation of the two ceramic restorations showed no significant difference. However, RNC restorations (Voco Grandio Blocks) obtained better scores than lithium disilicate ceramic restorations (IPS e.max) (Table 4). These findings align with those of previous clinical studies [12, 13, 36, 49]. The marginal adaptation of indirect restorations is influenced by various factors, including restorative materials, luting cement space (as mentioned earlier), and impression techniques [50, 51]. Several studies have demonstrated the superiority of RNC over disilicate lithium glass–ceramic restorations, which can be attributed to the smooth interface between indirect resin restoration and resin cement. Because of their similar mechanical properties, this smooth interface remains intact without degradation [13, 52].

Tsitrou et al. [53] have also suggested that the margins of resin-bonded restorations exhibit greater homogeneity than ceramic restorations, which aligns with the rationale behind the current study. Conversely, in a laboratory study, Yildirim et al. [8] reported lower marginal adaptation for RNC restorations (Lava Ultimate) and hybrid ceramic restorations (Vita Enamic) than for glass–ceramics (IPS e.max). These findings appear to contradict the results of the present study. Yildirim et al. [8] used a CEREC MC XL clinical-type milling unit with a 1.2-mm-diameter rotary instrument in their laboratory study. However, smaller-diameter rotary instruments are recommended to capture finer curvature details and achieve more accurate results. Additionally, other variables, such as the virtual space configuration in the software, intrinsic properties of the CAD/CAM system, and speed of the rotary milling instruments, may also affect the outcomes [7]. The decline in marginal adaptation observed for both restorations during the follow-up periods in the current study can be attributed to reliance on conventional impression techniques rather than full digital fabrication.

Marginal discoloration has been consistently observed in previous clinical studies [2, 28, 54] and is considered a common phenomenon. However, it has been determined that this discoloration does not affect the clinical performance of ceramic restorations [1]. The occurrence of marginal discoloration in both restorations was attributed to the deterioration of marginal adaptation during the follow-up period. Spitznagel et al. [55] also supported this assumption in their study, concluding that a decrease in marginal adaptation leads to increased marginal discoloration over time. Additionally, Frankenberger et al. [2] proposed an alternative hypothesis, suggesting that marginal staining could result from using self-etch and self-adhesive resin cements, which cannot etch the enamel surface. Furthermore, a separate microbiological study noted that salivary pellicles and dental biofilms could attach to surface irregularities at the tooth and restoration interface in patients with poor oral hygiene, causing marginal staining [56].

Regarding the color match, the present study revealed no significant differences between the two types of restorations. After a 2-year clinical follow-up, the evaluation indicated that only 61% of IPS e.max CAD and Voco Grandio restorations were rated as ALFA (A), as shown in Table 4. The significant color change within Voco Grandio group can be attributed to glaze loss after two years of clinical service. Staining is employed to enhance the aesthetic appearance of the restoration, providing a more natural look to the patient, and ultimately increasing patient satisfaction with the treatment. Glaze application is recommended to protect the stained layer [22].

In the case of glass–ceramics, stains can be effectively applied to restoration by subjecting them to high temperatures during firing. Depending on the ceramic material used, the staining process can occur before or after the crystallization/sintering firing cycle. However, staining and glazing are different processes for resin matrix ceramics, such as methyl methacrylate (MMA) light-cured composites [22].

To enhance the durability of glaze and stain layers, it is essential to prepare the surface of ceramic restorations by treating them with hydrofluoric acid etching or sandblasting using aluminum oxide particles. Following this surface preparation, applying a silane agent is recommended before glazing [22]. However, it is essential to note that these surface pretreatment steps were not performed in this study. Glazing was conducted in the laboratory without ceramic surface preparation.

Logically, this provides a clear justification for the early degradation of color and premature loss of the glaze layer, particularly in the Voco Grandio group in the current study. Jain et al. [57] also asserted that the glaze layer might be disrupted by acidic beverages, leading to the retention of stains and loss of color matching. In a laboratory study, Serrado de Pinho Barcellos, A., et al. [58] reported that staining and glazing of lithium disilicate glass–ceramic surfaces not only increased surface wear and bacterial adhesion but also decreased the biaxial flexural strength of the material. However, the validity of this assumption regarding glass–ceramic and RNC restorations requires further evaluation in future research.

Regarding surface texture, no statistical difference in surface roughness was detected between the restorative materials after 2 years of clinical follow-up. However, a significant difference was observed within each group over time, which could be attributed to all restorations in the posterior area, high occlusal forces, and occlusal adjustments performed after cementation. Mörmann et al. [23] reported that resin ceramic materials exhibited higher surface roughness than glass–ceramic materials. Additionally, Spitznagel et al. [55] reported increased surface roughness for hybrid ceramics in stress-functional areas. Restoration polishing, particularly for RNC restorations, appears to be a crucial step in maintaining long-term surface texture stability.

Regarding fractures, the present study found no statistically significant differences between the two indirect restorative materials, which aligns with various published studies [12, 13, 59]. Souza et al. [13] reported a case of chipping in the marginal ridge of a single restoration restored using lithium disilicate. However, this case did not require a complete replacement. Additionally, Aslan et al. [60] reported a minor fracture in a single case restored with a disilicate lithium restoration after one year of clinical follow-up, which did not necessitate a total replacement.

Regarding postoperative sensitivity, no significant difference was found between the two restorative materials, while there were significant differences between the baseline and 2-year follow-up within both ceramic material groups. Additionally, two cases involving RNC restorations exhibited intolerable postoperative hypersensitivity and were rated as Charlie (C) after one year of clinical follow-up. Santos et al. [54] suggested that removing all carious lesions, restoring deep dentin loss, and sealing all undercuts with resin-modified glass ionomer could minimize postoperative sensitivity. However, this approach was not used in this study. All carious lesions, defects, and weak margins were surgically removed, and the cavity design considered these factors during the restoration process using the selected indirect restorations. The postoperative hypersensitivity observed in the current study may be attributed to the inability to deliver the restoration at a single appointment, which could lead to tooth contamination during temporization and hinder proper bonding to freshly prepared tooth structures [15, 61, 62].

A strong and reliable bond between the restorative material and the luting agent is a crucial factor that significantly affects the long-term success of restorations. To achieve a robust bond, the recommended protocol for the internal surface conditioning of glass–ceramic restorations involves treating them with hydrofluoric acid, followed by a silane coupling agent [63, 64]. Whereas for RNC restorations, it is believed that the optimal protocol for enhancing bond strength outcomes involves mechanical sandblasting of the internal surface, followed by applying a silane coupling agent [15, 65]. In the present study, two debonded lithium disilicate ceramic restorations might be attributable to the inability to lute the restoration in a single appointment, which increases the risk of tooth contamination during temporization and compromises bond strength [61, 62].

The modified USPHS criteria have been widely regarded as reliable and standard methods for evaluating the clinical performance of ceramic restorations in various published literature [12, 35]. Moreover, a previous clinical study over ten years assessed the clinical performance of 200 feldspathic ceramic inlay and onlay restorations using the modified USPHS criteria [66]. This study provides a solid rationale for employing the modified USPHS criteria in the current clinical evaluation. However, it is worth noting that several published studies have suggested that the modified USPHS criteria are less practical and less relevant, with limited sensitivity and categories that may not comprehensively reflect the clinical success of restorations compared to the FDI criteria [67, 68].

In general, the findings of the present study demonstrated that lithium disilicate and RNC restorations exhibit satisfactory clinical performance, which aligns with the results of previous studies [11–13, 18, 20, 36]. A recent systematic review also emphasized that indirect resin-based composite restorations are dependable materials for partial-coverage restorations, with clinical performance comparable to that of glass–ceramic restorations [15].

At this level of study, it is essential to acknowledge the major limitations of the present investigation, which should be considered in future research. The primary limitation of the current study was its small sample size, which could be attributed to the challenges encountered in identifying patients with bilateral indirect ceramic restoration needs and good oral hygiene. Polishing cavity margins using rubber cups to ensure surface smoothness may compromise margins and complicate automated detection using CAD/CAM technology. Therefore, it is imperative to avoid this step in future studies. Another significant limitation of the current study is the inability to perform restoration at a single appointment, as previously mentioned. Additionally, using traditional impressions instead of digital or optical scanners adversely affects marginal adaptation.

## Conclusion

In the context of the current study, it is evident that IPS e.max CAD and hybrid RNC exhibit nearly identical clinical performance when evaluated over a 2-year period as per the modified USPHS criteria. However, additional clinical trials with extended follow-up periods are necessary to provide a more comprehensive understanding of the performance of these materials in community settings.

## Data Availability

The datasets used and analyzed in the current study are available from corresponding author on reasonable request.
